# Nutrient ileal digestibility evaluation of dried mealworm (*Tenebrio molitor*) larvae compared to three animal protein by-products in growing pigs

**DOI:** 10.5713/ajas.18.0647

**Published:** 2018-10-29

**Authors:** J. S. Yoo, K. H. Cho, J. S. Hong, H. S. Jang, Y. H. Chung, G. T. Kwon, D. G. Shin, Y. Y. Kim

**Affiliations:** 1 Daehan feed Co., Ltd., R&D Center, Incheon 22300, Korea; 2 Department of Agricultural Biotechnology, College of Animal Life Sciences, Seoul National University, Seoul 08826, Korea; 3 Jeonbuk Institute for Food-Bioindustry, Jeonju 54810, Korea; 4 Berry & Biofood Research Institute, Jeonbuk, Gochang, 56417, Korea

**Keywords:** *Tenebrio molitor*, Ileal Amino Acid Digestibility, Growing Pig

## Abstract

**Objective:**

This study was to investigate the nutrient ileal digestibility of dried mealworm (*Tenebrio molitor*) larvae and compare with those of three animal protein by-products in growing pigs.

**Methods:**

A total of 12 crossbred ([Landrace×Yorkshire]×Duroc) growing pigs with average body weights of 24.12±0.68 kg were surgically equipped with simple T-cannulas after being deprived of feed for 24 h according to published surgical procedures. These pigs had a recovery period of two weeks. A total of 12 pigs were assigned to individual metabolic crates and allotted to one of four treatments with 3 replicates in a fully randomized design. Dietary treatments included the following: i) Fish meal, corn-vegetable by-product basal diet+9.95% fish meal; ii) Meat meal, corn-vegetable by-product basal diet+9.95% meat meal; iii) Poultry meal, corn-vegetable by-product basal diet+9.95% poultry meal; iv) *Tenebrio molitor*, corn-vegetable by-product basal diet+9.95% dried *Tenebrio molitor* larvae.

**Results:**

Results showed that the apparent ileal digestibility (AID) of Lys was higher (p<0.05) in pigs fed *Tenebrio molitor* diet than that in pigs fed fish meal diet. Pigs fed *Tenebrio molitor* diet showed increased (p<0.05) AID of His and Arg compared to pigs fed Fish meal or Meat meal diet. The AID of Cys was increased (p<0.05) in pigs fed poultry meal and *Tenebrio molitor* diets compared to that in pigs fish meal diet. Pigs fed meat meal, poultry meal, and *Tenebrio molitor* diets showed higher (p<0.05) standardized ileal digestibility (SID) of total energy compared to pigs fed fish meal diet. The SID of Arg was higher (p<0.05) in pigs fed *Tenebrio molitor* diet than that in pigs fed fish meal or meat meal diet. Furthermore, pigs fed poultry meal or *Tenebrio molitor* diets showed increased (p<0.05) SID of Cys compared to pigs fed fish meal diet.

**Conclusion:**

In conclusion, providing pigs with diets that contained *Tenebrio molitor* larvae meal improved AID and SID of nutrients as well as essential and non-essential amino acids. The digestibility of dried mealworm larvae protein and its utilization *in vivo* are also good. Therefore, dried mealworm larvae protein can be used as protein source at 10% level in growing pigs.

## INTRODUCTION

The world population is expected to reach 9 billion in 2050 and production demand of meat and milk are expected to be 58% and 70% higher in 2050 than their levels in 2010 [1,2]. Increasing global population and meat consumption have led to urgent need for additional supplies of protein to be included in livestock feed. However, world’s agricultural land area is about 5 billion hectares, which is about 37% of the planet’s area. The area suitable for the production of crops, feed, and food is only 1.35 billion hectares. There is a limit to increase the production of crops to meet the increasing requirement of vegetable protein raw materials (soybean meal etc.) for livestock. Marine overexploitation has reduced the abundance of small pelagic forage fish from which fish meal and fish oil are derived for aquaculture feed. Prices of soybean meal and fish meal as traditional protein feed stocks will rise sharply which will limit their usage in the future.

To have sustainable protein-rich sources for aquaculture and livestock, one approach is to use insects as protein source. Both the scientific community and food and feed industrial sectors have begun to reconsider the use of insects as an alternative protein source as insects can be efficiently grown on organic side stream [3]. Such researches have so far focused on five major species or species groups [1,4,5]: the common housefly (*Musca domestica*), the black soldier fly (*Hermetia illucens*), the yellow mealworm (*Tenebrio molitor*), locusts (*Locusta migratoria*, *Schistocerca gregaria*, Oxya spec., *etc*.) and silkworms (*Bombyx mori*, *etc*.).

Insects are rich in proteins, fats, minerals, and vitamins. Their protein has a higher utilization rate than proteins of other animal by-products, thus attracting attention as a substitute for plant protein source [6,7]. In general, proteins contents in insects are 40% to 65%, similar to protein contents of meat meals. Lipid contents in insects (up to 36% oil) are also similar to lipid contents of meat meals. Unsaturated fatty acid concentration varies according to insect species: 60% to 70% in housefly maggot meal, yellow mealworm, and house cricket; 19% to 37% in black soldier fly larvae [4]. It is characterized by high lauric acid (C12:0) content, especially in black soldier fly larvae (*Hermetia illucens*). Some of them contain large amounts of zinc and iron [8]. However, most insect meals are deficient in Ca. In addition, insects can accumulate or secrete antimicrobial peptides in their body as a defense mechanism against pathogenic microorganisms [9]. It is believed that insects contain chitin which can enhance the immune function of livestock [10]. Makkar et al [4] have confirmed that palatability of insect larvae meal or insect meal to animals is good. Thus, insect larvae meal or insect meal could be used to replace 25% to 100% of soymeal or fishmeal depending on animal species.

Dried mealworm larvae contain high amounts of crude protein (CP, 46% to 52%) and fat (25% to 35%) which contain abundant essential fatty acids with superior oxidative stability [11]. Fresh larvae contain about 60% water. They have low Ca content and very low Ca:P ratio as other insects. Their Ca is slightly less available than Ca from oyster shells [12]. Exclusive feeding of mealworms can lead to Ca deficiency and symptomatic metabolic bone disease [12] because of their low Ca content and low Ca:P ratio. Studies on the feed value of the mealworm larvae have been done in poultry [4,13–17] and swine [18,19], but the results of the study were not enough.

Although information about the effect of mealworm feeding is insufficient, previous studies have indicated that insect larvae powder is a potentially promising protein source. Therefore, the objective of this study was to investigate the nutrient ileal digestibility of dried mealworm larvae in growing pigs compared to three animal protein by-products.

## MATERIALS AND METHODS

### Experimental animal and design

A total of 12 crossbred ([Landrace×Yorkshire]×Duroc) growing pigs with initial average body weight of 24.12±0.68 kg were surgically equipped with simple T-cannulas after being deprived of feed for 24 h according to surgical procedures of Stein et al [20]. These pigs had a recovery period of two weeks. A total of 12 pigs were assigned to individual metabolic crates and allotted to one of four treatments with 3 replicates in a fully randomized design. Dietary treatments included the following: i) Fish meal, corn-vegetable by-product basal diet+9.95% fish meal (TripleNine, Esbjerg, Denmark); ii) Meat meal, corn-vegetable by-product basal diet+9.95% meat meal (DK O&T, Eumseong, Korea); iii) Poultry meal, corn-vegetable by-product basal diet+9.95% poultry meal (Harim Co, Ltd., Iksan, Korea); and iv) *Tenebrio molitor*, corn-vegetable by-product basal diet+ 9.95% *Tenebrio molitor* (mealworm) larvae (3.5-month old, Daehan Feed Co, Ltd., Incheon, Korea). Three animal protein sources are commonly used in the livestock feed industry and the mealworms were harvested until about 3.5-month of age and obtained from Daehan feed’s insect experimental farm (Yesan, Korea). *Tenebrio molitor* (mealworm) larvae were dried by a hot-air drier (KEIL-2000, Keil, Seoul, Korea) and ground wholly by grinder for mealworm powder type. Room temperature was maintained at 27°C.

Chemical constituents of fish meal, meat meal, poultry meal, and dried mealworm (*Tenebrio molitor*) larvae are shown in Table 1.

### Experimental diet and feeding

Corn-vegetable by-product-based experimental diets were formulated to contain fish meal, meat meal, poultry meal, and dried mealworm (*Tenebrio molitor*) larvae powder at level of 9.95%, respectively. Chromic oxide was mixed to the diet at 0.5% as an indigestible marker to calculate digestibility. Metabolizable energy, CP, methionine, lysine, Ca, and P requirement of experimental diets were adjusted according to requirements of NRC [21,22]. Raw materials, chemical composition, and amino acid (AA) composition of experimental diets are shown in Table 2 and 3. Raw materials and chemical composition of nitrogen-free diets are shown in Table 4. For N-free diets, protein level was adjusted to the minimum with tapioca starch as base. Each pig was fed 1 kg of experimental diet four times a day at 07:00, 11:00, 15:00, and 19:00 h which was 2.8 times the maintenance energy requirement (MEn = 106 kcal ME/kg^0.75^ [21]). Pigs were provided free access to water at any time via a drinking nipple.

### Sample collection

Samples of ileal digested content were collected between 8:00 and 20:00 h for three days after five days of adaptation. Ileal digesta were collected into plastic bags attached to the cannulas and were emptied into a plastic containers containing ice every 20 minutes. All samples were immediately transferred after sampling and stored in a −60°C deep freezer to prevent changes in AA sequence due to microbes until analysis. Samples were dried in a freezing dryer and finely ground to pass through a 1-mm screen for chemical analysis including moisture, CP, and AAs contents.

### Chemical analysis

Diets and collected samples were grounded by a Cyclotec CT 193 Sample Mill (Foss Tecator, Hillerod, Denmark) and then analyzed. Analysis of the dry matter (DM) was conducted according to AOAC Method 967.03 [23]. The nitrogen content was analyzed by using the Kjeldahl procedure with Kjeltec (Kjeltec TM2200, Foss Tecator, Höganäs, Sweden) and calculating the CP content (Nitrogen×6.25; procedure 981.10; AOAC [23]). For the analysis of AA except for methionine and cysteine, diets and samples were hydrolyzed in 6 N HCl at 110°C for 24 hours (AOAC Method 999.13 [23]). Methionine and cystine were determined after cold performic acid oxidation overnight and hydrolyzed with 7.5 N HCl (AOAC method 994.12 [23]). Individual AA was measured by using an AA analyzer (Beckman 6300 Amino Acid Analyzer; Beckman Instruments Corp., Palo Alto. CA, USA). Chromium concentrations were determined via UV absorption spectrophotometry (UV-1201, Shimadzu, Kyoto, Japan). Gross energy was determined using a Parr 6100 oxygen bomb calorimeter (Parr instrument Co., Moline, IL, USA).

### Calculations

Standardized ileal digestibility (SID) of AA was calculated according to the method described by Stein [24,25] as an objective indicator of AA digestibility. Basal endogenous losses of CP and AAs were measured using samples collected at the end of the ileum after feeding with non-nitrogen feed [26]. Calculation of digestibility was done based on the relative chromium concentration of feed and ileal samples. AID and SID were calculated using the following equations:

$$\begin{array}{l} \text{Apparent ileal digestibility }(\text{AID},\%)=100-[(\text{ND}/\text{NF})\times (\text{CrF}/\text{CrD})\times 100]\\ \text{Basal endogenous AA losses }(\text{EAL})=[\text{ND}\times (\text{CrF}/\text{CrD})]\\ \text{SID }(\%)=[\text{AID}+(\text{EAL}/\text{NF})]\times 100 \end{array}$$

Where, ND, NF, CrF, and CrD were nutrient concentration in the ileum sample, nutrient concentration in the feed, chromium concentration in the feed, and chromium concentration in the ileum sample, respectively.

### Statistical analysis

Each pig was considered as experimental unit. All data were analyzed using the general linear model procedure of SAS (SAS Inst. Inc., Cary, NC, USA). Results were compared by the least significant difference multiple test method. Differences among treatment means were determined using Tukey’s test with significance level at p<0.05.

## RESULTS

Nutrient contents of dried mealworm larvae and the other three animal protein sources are summarized in Table 1. These data indicated that mealworm larvae contained higher percentage of essential AAs (46.3%) than other protein sources. Results of AID are presented in Table 5. The AID of Lys was higher (p<0.05) in pigs fed *Tenebrio molitor* diet compared to that in pigs fed Fish meal diet. Pigs fed *Tenebrio molitor* diet showed increased (p<0.05) AID of His and Arg compared to pigs fed Fish meal or Meat meal diet. In addition, the AID of Cys was increased (p<0.05) in pigs fed Poultry meal or *Tenebrio molitor* diet compared to pigs fed Fish meal diet. Although there were no significant differences, pigs fed *Tenebrio molitor* diet tended to show increased AID of DM, total energy, CP, total AAs, and the rest of essential AAs (Met, Thr, Val, Ile, Leu, and Phe) and non-essential AAs (Asp, Ser, Glu, Gly, Ala, Tyr, and Pro) than pigs fed fish meal, meat meal, or poultry meal diet. The digestibility of each nutrient showed similar tendency in the case of poultry meat and meat meal diets.

Pigs fed meat meal, poultry meal, or *Tenebrio molitor* diet had higher (p<0.05) SID of total energy compared to pigs fed fish meal diet (Table 6). The SID of Arg was higher (p<0.05) in pigs fed *Tenebrio molitor* diet compared to that in pigs fed fish meal or meat meal diets. Furthermore, pigs fed poultry meal, meat meal, or *Tenebrio molitor* diet showed increased (p<0.05) SID of Cys compared to pigs fed fish meal diet. Pigs fed with *Tenebrio molitor* diet tended to show increased SID of DM, total energy, CP, total AAs, the rest of essential AAs (Lys, Met, Thr, Val, Ile, Leu, Phe, and His), and the rest of non-essential AAs (Asp, Ser, Glu, Gly, Ala, Tyr, and Pro) than pigs fed meat meal, poultry meal, or fish meal diet.

## DISCUSSION

In general, dried mealworm larvae contain high amounts of CP (46% to 52%) and fat (25% to 35%) with relatively low ash content [4,27]. Fresh larvae contain about 60% moisture. They are also relatively low in Ca content. The dried mealworm larvae used in this study were about 3.5-month old with CP, essential AA, crude fat, and Ca contents of 48.2%, 46.3(g/16 g-N), 29.5%, and 0.04%, respectively. The AA profiles and protein contents of dried mealworm larvae and three animal protein by-products are superior to soybean meal, the main protein source used in pig and poultry feed. Soybean meal has a CP content of 49% to 56% DM and has a crude fat content of 3% DM [28]. The other three animal protein sources have protein content of 65.5% to 73% in as-fed basis. With regard to protein content and AA quality, dried mealworm larvae are comparable to meat meal and poultry meal as well as fish meal. The other three animal protein sources have protein content of 65.5% to 73% in as-fed basis. Essential AA (lysine, methionine+cystine, threonine and tryptophan) index, EAAI [29] values for fish meal, meat meal, poultry meal and dried mealworm larvae were 0.57, 0.40, 0.45, and 0.58, respectively (AA requirements based on Daehan feed Co. data). EAAI of dried mealworm larvae was similar to that of fish meal.

Fat content of animal protein source in this experiment was 9.71% for fish meal, 11.09% for meat meal, 13.00% for poultry meal, and 29.5% for dried mealworm (*Tenebrio molitor*) larvae. Because experimental diets were formulated with the same energy and dried mealworm (*Tenebrio molitor*) larvae had the highest fat content and the lowest ash content, *Tenebrio molitor* diet had lower ground corn content and higher wheat bran content resulting in higher crude fiber content than the other three diets. This might can have an impact when designing grower feed formula and feeding the diet to grower. Although excessively high fiber content of diet limits feed intake, adequate fiber content can have a beneficial effect on the environment of large intestine. Previously studies have measured CP digestibility of some insect larvae [19,30–32]. Newton et al [31] have reported that the apparent faecal digestibility of black soldier fly larvae in male growing pigs is 76% similar apparent CP digestibility as soybean meal. Jin et al [19] have shown that the total track digestibility of CP is linearly increased with increasing dried mealworm level. The digestibility in their studies was 92.17% (level of dried mealworm larvae included in the diet was 4.5%) or 93.04% (level of dried mealworm larvae included in the diet was 6.0%). That of our experiment is higher than that of Newton et al [31] and lower than that of Jin et al [19]. In general, the apparent total track protein digestibility is lower than the ileal digestibility due to microorganisms in the large intestine. In addition, the age of the experimental animals [31] was 5 weeks at which the development of protein digestion enzymes was not sufficient. Although the protein digestibility of Jin et al [19] was a total track digestibility and the pigs were younger than those of this study, that was higher than that of this experiment due to experimental diet composition (mainly soybean meal was used as vegetable protein by-product) expected to have a higher digestibility than palm kernel meal and wheat bran, and lower feeding amount (2% of body weight) than that of this study (4% of body weight).

The protein digestibility of the dried mealworm larvae containing diets tended to be slightly higher than those of the other experimental diets containing other animal by-products (p = 0.05). Increased dietary protein digestibility aids weight gain in chicks [30] and weaning pigs [19]. Although growth performance was not determined in this study, improved growth performance with dried mealworm larvae could be deduced with current result of protein digestibility when compared with other three animal by-products.

Hwangbo et al [30] and Pretorius [32] have conducted studies using housefly meal in broilers and reported digestibility of 98.5% and 69%, respectively. However, the later study reported that the AA digestibility was over 90% while the CP digestibility was much lower. This might have attributed to indigestibility of chitin-N and/or ADF bound-N [32]. Chitin, a linear polymer of β-(1–4) N-acetyl-D-glucosamine units with a chemical structure similar to that of cellulose, is combined with protein and distributed widely on cell wall and shell of crabs, shrimp, and insects. Chitin is rarely found in nature alone. It is distributed in the form of chitin-protein complexes as a major component of the cuticle layer [33]. In addition, proteins of *Tenebrio molitor* larvae are mainly composed of proteins derived from cuticle and other proteins, including various hemolymph proteins, endurance proteins (such as hexamerin), and different enzymatic proteins [34]. In this study, the protein digestibility was relatively high (AID, 89.58%; SID, 90.05%) and there was no significant difference when compared with those of total AA digestibility (AID, 89.60%; SID, 90.05%). There was little effect of chitin on protein digestibility. The chitin content of mealworm larvae has been estimated to be 2.8% of DM [35]. Although dried mealworm larvae have protein-chitin complex, the inclusion rate of that was only 9.95% in total experimental diet. It had little effect on digestibility of total feed protein. It can be deduced that there is no need to consider protein-chitin complex at about 10% level as a protein source.

Pigs fed *Tenebrio molitor* larvae meal showed similar AID of lysine (89.65%), histidine (89.68%), and arginine (89.74%) with results of Ji et al [18], reporting that AID values of lysine, histidine, and arginine were 89.33%, 88.00%, and 92.00%, respectively, in early-weaned piglets (Phase 2, Day 29 to 56). Even though the pigs were younger than those of this study, the digestibility was similar due to complexity of experimental diet, having more digestible raw materials. However there was significant variation in AID values of alanine, cystine, and glycine (78.67%, 77.00%, and 70.67%, respectively) in the results of Ji et al [18] while there was no significant variation in digestibility between AAs in this study. They inferred that the lower AID values were due to different AA structure and said that further studies were needed to explore this possibility [18]. Pigs fed *Tenebrio molitor* larvae meal showed higher AID and SID of lysine (89.65% and 89.96%), histidine (89.68% and 90.07%), and arginine (89.74% and 89.9%) in comparison with pigs in other treatment groups.

It would appear from the results of this study, that dried mealworm (*Tenebrio molitor*) larvae would be a suitable ingredient in growing pigs, being especially valuable from the stand-point ileal AA digestibility. High protein and AA digestibility of dried mealworm larvae may act on the deposition of AAs in pig meat and on the growth performance in growing pigs. Further studies are needed to explore this possibility.

## CONCLUSION

In conclusion, the nutritional value of dried mealworm larvae closely matched with that of fishmeal, making it a potentially attractive alternative protein-rich feed ingredient for livestock feed industry. Dietary supplementation of dried mealworm larvae had higher digestibility in DM, CP, total AAs, essential AAs, and non-essential AAs compared to dietary supplementation with fish meal, meat meal, or poultry meal. Thus, dried mealworm larvae protein can be used as a protein source at 10% level in growing pigs.

## Figures and Tables

**Table 1:** Composition of fish meal, meat meal, poultry meal and dried mealworm larvae

Chemical composition (%)1)	Fish meal	Meat meal	Poultry meal	Dried mealworm larvae
Moisture	8.0	4.0	4.0	5.9
Crude protein	69.0	73.0	65.5	47.8
Crude fat	8.5	10.0	13.0	34.6
Crude fiber	0.8	0.5	2.0	6.1
Crude ash	15.0	15.0	12.8	6.3
Ca	3.20	5.00	3.40	0.05
Total P	2.30	2.40	2.10	0.59
Amino acid2)				
Essential	29.19	21.31	24.55	42.30
Methionine	1.86	1.00	1.38	1.34
Cystine	0.62	0.44	0.59	0.86
Valine	3.31	2.70	2.95	4.98
Isoleucine	2.76	1.83	2.36	3.76
Leucine	5.04	3.80	4.45	6.88
Phenylalanine	2.70	2.20	2.42	3.72
Tyrosine	2.20	1.61	2.09	7.76
Histidine	2.07	1.31	1.38	2.93
Lysine	5.18	3.65	3.86	5.42
Threonine	2.69	2.26	2.55	4.04
Tryptophan	0.76	0.51	0.52	0.61
Non-essential	32.79	43.74	35.70	48.65
Serine	2.69	2.63	2.55	4.83
Arginine	3.80	5.04	4.39	5.46
Glutamic acid	8.63	8.30	8.45	11.54
Aspartic acid	6.42	5.69	4.30	8.01
Proline	3.04	6.24	5.65	6.73
Glycine	4.00	10.51	6.03	5.06
Alanine	4.21	5.33	4.52	7.02

1) Lab. of Daehanfeed Co. LTD.

2) g/16 g nitrogen.

**Table 2:** Composition of the experimental diets, as-fed basis

Items	Treatments			

	Fish meal	Meat meal	Poultry meal	*Tenebrio molitor*
Ingredients (%)				
Ground corn	73.89	73.59	75.89	63.27
Soybean meal, 45%	5.14	4.69	5.65	9.35
Fish meal	9.95	0	0	0
Meat meal	0	9.95	0	0
Poultry meal	0	0	9.95	0
Meal worm	0	0	0	9.95
Palm kernel meal	2.98	3.00	2.99	2.99
Wheat bran	4.64	6.14	1.90	10.42
Tallow	1.16	1.17	1.17	0.67
MDCP	0.48	0	0.53	0.94
Limestone	0.66	0.03	0.61	1.07
DL-methionine, 99%	0	0.09	0.05	0.10
L-lysine-HCl, 78%	0.13	0.34	0.27	0.24
Salt	0.30	0.30	0.30	0.30
Vitamin premix1)	0.10	0.10	0.10	0.10
Mineral premix2)	0.10	0.10	0.10	0.10
Cr_2_O_3_	0.50	0.50	0.50	0.50
Sum	100	100	100	100
Chemical composition3)				
ME (kcal/kg)	3,249	3,249	3,249	3,249
Crude protein (%)	15.50	15.50	15.50	15.50
Crude fat (%)	4.52	4.71	4.97	6.01
Crude fiber (%)	2.61	2.67	2.37	3.70
Crude ash (%)	5.21	4.16	4.93	5.62
Lysine (%)	0.95	0.95	0.95	0.95
Methionine (%)	0.35	0.35	0.35	0.35
Ca (%)	0.66	0.66	0.66	0.66
Total P (%)	0.56	0.58	0.56	0.56

MDCP, mono-di-calcium phosphate (Ca, 17.6% and total P, 20.4%).

1) Provided the following per kilogram of diet: vitamin A, 7,960 IU as vitamin A acetate; vitamin D_3_, 1,592 IU; vitamin E, 32 IU as dl-α-tocopheryl acetate; biotin, 0.25 mg as d-biotin; riboflavin, 3.2 mg; thiamine 3 mg as thiamine mononitrate; pyridoxine 5 mg as pyridoxine hydrochloride; folic acid 0.5 mg; pantothenic acid, 8 mg as d-calcium pantothenate; niacin, 46 mg as nicotinic acid; vitamin B_12_, 12 μg as cyanocobalamin; and vitamin K_3_, 2.4 mg as menadione nicotinamide bisulfate.

2) Provided the following per kilogram of diet: Cu 24.8 mg as CuSO_4_H_2_O; Fe 54.1 mg as FeSO_4_H_2_O; Zn 84.7 mg as ZnSO_4_H_2_O; Mn 24.8 mg as MnSO_4_H_2_O; I 0.3 mg as Ca(IO_3_)H_2_O; Co 0.3 mg as CoSO_4_H_2_O; and Se 0.1 mg as NaSeO_3_H_2_O.

3) Calculated values.

**Table 3:** Amino acid composition of experimental diets

Items	Treatments1)				SEM

	Fish meal	Meat meal	Poultry meal	*Tenebrio molitor*	
Total amino acid (%)2)	15.07	13.40	15.19	16.09	0.971
Essential amino acid (%)3)					
Lys	0.98	0.88	1.10	1.21	0.124
Met	0.28	0.28	0.23	0.29	0.023
Thr	0.71	0.66	0.72	0.76	0.036
Val	0.60	0.57	0.69	0.73	0.065
Ile	0.56	0.54	0.67	0.71	0.072
Leu	1.22	1.10	1.24	1.32	0.079
Phe	0.75	0.64	0.74	0.79	0.055
His	0.38	0.34	0.39	0.41	0.025
Arg	1.03	0.88	1.04	1.10	0.081
Non-essential amino acid (%)3)					
Asp	1.66	1.41	1.63	1.68	0.108
Ser	0.83	0.71	0.80	0.83	0.049
Glu	2.75	2.38	2.71	2.87	0.182
Gly	0.77	0.67	0.74	0.78	0.043
Ala	0.83	0.76	0.81	0.85	0.033
Tyr	0.44	0.37	0.44	0.48	0.040
Pro	1.04	0.97	1.03	1.05	0.031
Cys	0.24	0.24	0.21	0.23	0.012

SEM, standard error of means.

1) Fish meal, corn-vegetable by-product based diet with 9.95% fish meal; meat meal, corn-vegetable by-product based diet with 9.95% meat meal; poultry meal, corn-vegetable by-product based diet with 9.95% poultry meal; *Tenebrio molitor*, corn-vegetable by-product based diet with 9.95% dried *Tenebrio molitor* larvae.

2) Total amino acid: Essential amino acid (%)+non-essential amino acid (%).

3) Chemical analysis values.

**Table 4:** Composition of the nitrogen-free diets, as-fed basis

Items	
Ingredient (%)	
Tapioca starch	67.25
Soy-oil	9.22
Sucrose	9.95
Lactose	9.95
MDCP	2.63
Limestone	0.00
Salt	0.30
Vitamin premix1)	0.10
Mineral premix2)	0.10
Cr_2_O_3_	0.50
Sum	100.0
Chemical composition3)	
ME (kcal/kg)	3,249
Crude protein (%)	0.00
Lysine (%)	0.00
Methionine (%)	0.00
Threonine (%)	0.00
Ca (%)	0.70
Total P (%)	0.60

MDCP, mono-di-calcium phosphate (Ca, 17.6% and total P, 20.4%); ME, metabolizable energy.

1) Provided the following per kilogram of diet: vitamin A, 7,960 IU as vitamin A acetate; vitamin D_3_, 1,592 IU; vitamin E, 32 IU as dl-α-tocopheryl acetate; biotin, 0.25 mg as d-biotin; riboflavin, 3.2 mg; thiamine 3 mg as thiamine mononitrate; pyridoxine 5 mg as pyridoxine hydrochloride; folic acid 0.5 mg; pantothenic acid, 8 mg as d-calcium pantothenate; niacin, 46 mg as nicotinic acid; vitamin B_12_, 12 μg as cyanocobalamin; and vitamin K_3_, 2.4 mg as menadion nicotinamide bisulfate.

2) Provided the following per kilogram of diet: Cu 24.8 mg as CuSO_4_H_2_O; Fe 54.1 mg as FeSO_4_H_2_O; Zn 84.7 mg as ZnSO_4_H_2_O; Mn 24.8 mg as MnSO_4_H_2_O; I 0.3 mg as Ca(IO_3_)H_2_O; Co 0.3 mg as CoSO_4_H_2_O; and Se 0.1 mg as NaSeO_3_H_2_O.

3) Calculated values.

**Table 5:** Effect of protein source on apparent ileal digestibility in growing pigs

Items	Treatments1)				SEM	p-value

	Fish meal	Meat meal	Poultry meal	*Tenebrio molitor*		
Dry matter (%)	84.35	86.99	87.65	89.44	2.679	0.08
Gross energy (%)	83.62	88.39	87.86	89.53	3.135	0.06
Crude protein (%)	85.04	86.76	87.87	89.58	2.387	0.05
Total amino acid (%)	85.37	86.90	87.97	89.60	1.543	0.05
Essential amino acid (%)						
Lys	86.13^b^	87.36^ab^	88.40^ab^	89.65^a^	1.296	0.04
Met	85.53	87.19	87.76	89.56	1.440	0.06
Thr	84.17	86.82	87.59	89.53	1.925	0.06
Val	84.34	85.99	87.88	89.51	1.946	0.05
Ile	84.07	86.25	87.87	89.38	1.971	0.05
Leu	85.05	86.41	87.89	89.59	1.691	0.05
Phe	85.66	86.88	87.98	89.59	1.448	0.06
His	85.51^b^	86.68^b^	88.28^ab^	89.68^a^	1.579	0.04
Arg	87.37^b^	87.66^b^	88.93^ab^	89.74^a^	0.961	0.03
Non-essential amino acid (%)						
Asp	85.66	87.10	87.96	89.60	1.427	0.05
Ser	85.14	87.03	88.04	89.61	1.622	0.06
Glu	86.44	87.47	88.34	89.72	1.202	0.06
Gly	84.22	86.39	87.14	89.53	1.896	0.06
Ala	83.64	86.23	86.9	89.45	2.066	0.06
Tyr	84.45	86.57	87.5	89.47	1.806	0.06
Pro	84.97	86.76	87.64	89.62	1.676	0.07
Cys	83.00^b^	86.02^ab^	87.64^a^	89.57^a^	2.406	0.04

SEM, standard error of means.

1) Fish meal, corn-vegetable by-product based diet with 9.95% fish meal; meat meal, corn-vegetable by-product based diet with 9.95% meat meal; poultry meal, corn-vegetable by-product based diet with 9.95% poultry meal; *Tenebrio molitor*, corn-vegetable by-product based diet with 9.95% dried *Tenebrio molitor* larvae.

ab Means in a same row with different superscript significantly different (p<0.05).

**Table 6:** Effect of protein source on standardized ileal digestibility in growing pigs

Items	Treatments1)				SEM	p-value

	Fish meal	Meat meal	Poultry meal	*Tenebrio molitor*		
Dry matter (%)	85.66	88.31	88.96	89.53	2.484	0.08
Gross energy (%)	83.30^b^	90.01^a^	88.76^a^	90.96^a^	3.339	<0.01
Crude protein (%)	85.55	87.32	88.38	90.05	2.270	0.06
Total amino acid (%)	85.85	87.44	88.44	90.05	1.527	0.06
Essential amino acid (%)						
Lys	86.51	87.78	88.73	89.96	1.264	0.05
Met	85.87	87.53	88.17	89.89	1.439	0.06
Thr	84.79	87.50	88.21	90.12	1.910	0.06
Val	85.02	86.71	88.47	90.07	1.891	0.06
Ile	84.77	86.98	88.46	89.93	1.908	0.06
Leu	85.45	86.85	88.28	89.96	1.675	0.06
Phe	86.01	87.29	88.34	89.93	1.436	0.06
His	85.94	87.15	88.69	90.07	1.561	0.05
Arg	87.61^b^	87.95^b^	89.17^ab^	89.97^a^	0.947	0.04
Non-essential amino acid (%)						
Asp	86.06	87.57	88.37	90.00	1.420	0.06
Ser	85.66	87.64	88.58	90.13	1.619	0.06
Glu	86.75	87.83	88.65	90.01	1.193	0.06
Gly	84.82	87.08	87.76	90.12	1.890	0.07
Ala	84.17	86.8	87.43	89.96	2.060	0.07
Tyr	84.84	87.03	87.89	89.83	1.791	0.06
Pro	86.08	87.96	88.77	90.73	1.667	0.07
Cys	83.62^b^	86.64^ab^	88.35^a^	90.21^a^	2.425	0.04

SEM, standard error of means.

1) Fish meal, corn-vegetable by-product based diet with 9.95% fish meal; meat meal, corn-vegetable by-product based diet with 9.95% meat meal; poultry meal, corn-vegetable by-product based diet with 9.95% poultry meal; *Tenebrio molitor*, corn-vegetable by-product based diet with 10% dried *Tenebrio molitor* larvae.

ab Means in a same row with different superscript significantly different (p<0.05).
